# Review of Referee Board on Benzoate of Soda1Delivered before the State and National Food and Dairy Commissioners at Denver, August, 26 1909,

**Published:** 1909-10

**Authors:** Charles A. L. Reed

**Affiliations:** Cincinnati, Ohio


### BUFFALO MEDICAL JOURNAL.

VOL. LXV. OCTOBER, 1909. NO. 3

### ORIGINAL COMMUNICATIONS.

### Review of Referee Board on Benzoate of Soda.[1]

BY CHARLES A. L. REED, M. D., Cincinnati, Ohio.

AFTER a long series of experiments conducted by the government through one of its bureaus designed by law for the purpose and following the enactment of the food and drugs act, articles of food preserved by benzoate of soda were excluded from interstate commerce as being deleterious to the public health.

THE COMMERCIAL INTERESTS.

This action on the part of the government was embarrassing to two distinct commercial interests—namely, the manufacturers of benzoate of soda and the manufacturers of food products of such inferior quality that they could not be preserved without the use of benzoate of soda. These interests protested to the government that the original experiments were either defective or at least not conclusive that benzoate of soda as a food preservative was not deleterious within the meaning of the law, and demanded a reinvestigation of the subject at the hands of an independent commission.

THE GOVERNMENT'S ACTION.

The government acceded to that demand and appointed several of the best known physiological chemists as a referee board of consulting scientific experts with instructions to determine whether or not a food to which benzoate of soda had been added in either large or small quantities contains, by virtue of that fact, "any added poisonous or other added deleterious ingredient which may render the said food injurious to health." This referee board then conducted, or caused to be conducted, a series of experiments at the conclusions of which it filed a report unreservedly favorable to benzoate of soda as a food preservative. The government being virtually precommitted to the findings of its own referees was thereby induced to reverse itself and on March 3,

1. Delivered before the State and National Food and Dairy Commissioners at Denver, August, 26 1909.

1909, practically the last act of the Roosevelt administration, issued a decision to the effect that no objection would be raised "to the use in food of benzoate of soda provided that each container or package of such food is plainly labeled to show the presence and amount of benzoate of soda."

### AN OMINOUS DECISION.

This decision is far reaching. It affects not only many millions of capital, but the health and lives of many more millions of people. It involves, furthermore, so serious a step as the reversal by a great government of its previously deliberately determined policy on this question. These facts make it inevitable that while the government must go without criticism as being justified by the report of its chosen referees, the report upon which so momentous a decision was based must become the object of critical scrutiny from every view point that may tend either to confirm or destroy its validity. And while in response to your invitation, I speak here today by virtue of my right as a citizen and a consumer to discuss this question, my special view points are, primarily, that of a physician, and, secondarily, that of an official of the great organised medical profession that stands and always must stand as the natural guardian of the public health—a profession which was largely instrumental in securing the enactment as it will labor for the protection and improvement of the legislation under which these various steps have been taken.

### SOME POINTED QUESTIONS.

An approach to the general question, from these view points, prompts several queries that are suggested by the referee's report itself:

(1) Did the questions submitted by the government cover the whole ground?

(2) Were the experiments or any of them actually conducted by the members of the Referee Board of Scientific Experts?

(3) Were the experiments (a) carried out with sufficient care, (b) were they sufficiently comprehensive and (c) were the demonstrated facts recorded and interpreted with sufficient freedom from bias to entitle the report, as a whole, to public confidence?

(4) Do the recorded facts justify the conclusions embodied in the report, upon which conclusions rather than upon the recorded facts, hidden as they are in an avalanche of irrelevant data, the government naturally relied as a basis for its action?

### WHAT IS BENZOATE OF SODA?

But before attempting an answer it is important to bear in mind that benzoate of soda is a salt resulting from the action of benzoic acid or sodium. As it occurs in our commerce it is derived from two sources—namely, Germany, where it is made from the urine of horses and cows, and from the United States, where it is made from coal tar. Its effects on the human system as determined by many pharmacologists—men skilled in determining the effects of drugs—are rather definite. Locally it is a mildly irritating antiseptic causing redness of the skin when applied to it, and irritation of the mucous membrane of the nose and throat with sneezing and coughing when snuffed as a powder. Taken internally it causes a sense of heat over the stomach and an increase in the frequency of the pulse associated in instances with fulness of head and slight vertigo. In full doses it causes nausea with vomiting, blood sometimes appearing in the vomited matter. It often causes diarrhea followed in some instances by constipation. It is eliminated from the system by the kidneys and converts alkaline urine into acid urine, while rendering acid urine less acid. It is used as an application to the skin to cure the itch, and to chronic ulcers to force them to heal. It is given as an intestinal antiseptic to destroy various germs. It is given to increase the quantity and change the quality of the urine. It is administered as a stimulating expectorant in certain coughs and as a disinfectant in diphtheria and scarlet fever, the dose being from 0.65 gram. to 1.30 gram. These facts recorded by the most scientific pharmacologists and clinicians, are confirmed by the unanimous practice of tens of thousands of medical practitioners all over the country. And let me add, that these same facts find their most recent and emphatic, although reluctantly given, confirmation at the hands of the Referee Board of Scientific Experts of the United States Department of Agriculture, whose report (No. 88) is now under review.

### SOME INTERESTING COMMERCIAL CONDITIONS.

Then, too, it is important to remember that benzoate of soda, by virtue of its properties as a powerful antiseptic, was used and, since the removal of the ban by the government, is again being used as a preservative for food. Experience amply proves that sound fruits and vegetables, including catsup from sound tomatoes, can be made to keep without the addition of benzoate of soda or of any other added deleterious medicament. Unsound fruit and the slush from canning factories, slush that ought to go only into the sewer, can be and are today being preserved, purveyed and extensively consumed as articles of food by the simple process of first medicating them with benzoate of soda, permission to do which is based upon the findings of this referee board. It is to be remembered that the utilisation of this material which, without the use of benzoate of soda, must go to waste, represents a large margin of profits to certain selfishly commercialised interests, and that this fact accounts in large part for the pressure brought upon the government to reverse its original position.

QUESTIONS DODGED BY THE REFEREES.

It must follow from what I have said that the government naturally desires the fullest possible information on the whole subject. This embraces not only the effect of the drug itself in large and small doses on the human system and on the nutritive value of foods subjected to its influence, which the board assumed to investigate, but other phases of the problem of the most vital character to which the board did not give the slightest attention. Thus one phase was the limit to which the benzoate could be added to foods without, as the board avers, in the slightest degree damaging their nutritive value. This is a point of extreme importance in view of the fact that, on the findings of these referees, the government has proclaimed a license to medicate the food of the people with this drug in any quantity with absolutely no restriction upon the articles of food that may be subjected to its use. Are we to understand that even these referees, partisans of benzoate that I shall show them to be, have the termerity to say that this powerful drug can be added to food in all proportions without impairing its nutritive value or that it can be eaten by the people, in all quantities, at all times and under all conditions without serious consequences to their health?

Then, too, it was manifestly incumbent upon this board to have determined the amount of benzoate required to preserve (a) sound food, (b) unsound food and (c) sewage. But it has seen fit also to ignore this practical phase of the problem in spite of the fact that these commercial usages must have been known to them, as shown by their discussion of other commercial usages, and in spite of the fact that the specific interrogatories submitted to them by the government logically embrace these and all other points necessary for its intelligent guidance in the solution of this great problem.

THE SCIENTISTS DID NOT DO THE WORK.

But did the scientists thus appealed to for the guidance of the government in dealing with the lives and health of millions of people, and who were appealed to largely because of the prestige their personal attention to the question and the universities with which they were connected would give to the final decision—I ask, did these scientists do any of the actual work involved in the solution of this momentous problem? So far as indicated by the report, which cost well on to $100,000, not a single analysis of medicament, food or secretion, not a single physical examination, not a single record, was made by any of the three eminent gentlemen whose names head this report. The whole thing seems to have been turned over to subordinates, doubtless talented if left unhampered, whose work be it said is recognised with praiseworthy frankness by the nominal referees. It seems indeed that the function of the referees has been to lay out the work, turn it over to their subordinates to do, and when it was done, to take the data thus accumulated and subject it to such construction as would best subserve some argumentative point in the interest of benzoate of soda, rather than the purposes of the government that employed them to render actual assistance in protecting the people against deleterious food.

### A FUNDAMENTAL FALLACY.

In framing its experiments upon which to base its reply to the interrogatories posed by the government, the referee board arranged that they should be conducted by each of three members—one set of experiments on six men in New Haven, one set on four men in New York and one set on six men in Chicago. The subjects were men, healthy and robust, some students, some physicians, and all, I believe, engaged in active pursuits, some of them athletes, the majority of them between 20 and 30 years old. None, I believe, was over 43. All, therefore, possessed the maximum power of resistance. In these facts we find the first as it is one of the most serious fallacies underlying the investigations; for the average index of resistance of sixteen robust young men can not be accepted as the average index of resistance of the young and old, the well and the ill, the strong and the weak who make up the promiscuous millions that are consumers of medicated foods. The well known difference between men and women in their respective susceptibility to drugs, recognised and acted upon by every intelligent physician in the land, is a fact that further accentuates the fundamental fallacy to which I have alluded and one that would hardly have escaped the referee board if it had been made up of pharmacologists who are in the habit of investigating such questions. It is obvious from these considerations that the question at issue is one that must appeal for answer to the accumulated experience of the medical profession rather than to any board, however expert, that relies exclusively upon defective laboratory methods for its results.

### A WONDERFUL DIET—SOME MORE FALLACY.

The subjects thus accepted were not only of the highest general average of resistance, but it would seem that their diet was arranged with reference to keeping that resistance at the maximum by giving them the benefits of forced nutrition. In New

Haven they had a dietary practically unlimited in variety and amount—at some meals some of the subjects taking as many as twenty-five different articles. In Chicago, "the men were allowed a very ample diet following their own tastes and desires as far as possible"—which, interpreted in the light of further information given would seem to have meant a sort of riot of the gourmands. In New York, it is only necessary to say that among the daily menus published was one that embraced oranges, cantaloupes, scrambled eggs, ham, bread, butter, sugar, milk, force, tomatoes, soup, stringbeans, mashed potatoes, fried potatoes, veal cutlets, gravy, metropolitan cake and coffee, aggregating 2405.3 grams of food. This was fairly typical of other menus which, however, in some instances, embraced the added attractions of sour pickles and bologna! What science can unravel the chemistry of such a mess which, weighed in without analysis at each meal, seemed to be limited only by the physical capacity of the stomach rather than digestive and assimilative capacity of the subjects? It had, however, the natural effect of maintaining both nutrition and the powers of resistance at the maximum during the comparatively short period of the experiments. At the same time by mixing, if not losing, the three-tenths of a gram of the benzoate in nearly two thousand five hundred grams of food the assimilation of the benzoate itself and, consequently, its effects were reduced to a minimum. Some of the diet, as for instance the excess of milk, seemed arranged with reference to its antidotal effects on the benzoate which, in certain instance, was doubtless thereby practically eliminated from the experiment.

AN EXAMPLE OF DISCREET EXPERIMENTATION.

This fact and some others equally important, become apparent when the manner of administering the benzoate of soda to these subjects is taken into account. "The salt was given three times a day—0.1 gram of benzoate with each meal—and in some one article of food where it would be present to the extent of about one-tenth of 1 per cent. by weight of food." This was continued for several weeks after which, in the New Haven group at least, there was a period of something more than a week of abstinence from the benzoate for the purpose of recuperation. Then the medicament was resumed by giving 0.6 gram, 1.0 gram, 2.0 grams and 4.0 grams in consecutive order each for a period of one week. This was followed by another week of abstinence from the benzoate after which the subject was weighed out of the ring. While there were some variations in details, practically the same methods were observed in all of the groups.

### A FEW MORE POINTED QUESTIONS.

Several more important queries are suggested by these facts In the first place, in view of the fact that "an attempt was made to imitate the manner in which the salt would be taken if used in food as a preservative," why was not 2.10 per cent. instead of 1.10 per cent. used as a basis of computation? And why was not the minimum dosage arranged accordingly? The query is pertinent in view of the fact that the larger percentage is the one frequently if not more generally employed in commercial preservation of food. And it becomes especially pertinent and the dosage employed by order of the referees becomes ridiculously small, if the license for the unlimited medication of food by benzoate of soda, already granted by the government, acting under the advice of this referee board, shall result in preparation of food in that manner up to the limit of possibility.

Then, too, it is an ascertained fact, familiar to physicians in general, that certain of the effects of benzoate of soda on the human system are cumulative in character. The accurate determination of this fact by the experiments under review was made impossible, the cumulative effects of the drug having been reduced to a minimum by the interval in administration for a week and by the omission of the drug from the food for another whole week before a final accounting was made of the subjects' condition. When our dietary shall have become entirely benzoated must the people from time to time go a whole week without food to maintain their normal index of resistance? And must they go still another week without food if they shall desire to determine their actual state of health? Fortunately for this phase of the question certain facts elicited by the actual experimenters and to which I shall subsequently allude, seem to have escaped the censorship of the referees and are now to be found, if searched for, in the body of the report.

### SOME STRANGE RULES OF EVIDENCE.

The effects on the human system of this mildly medicated diet, dictated by the referees, and not the experimenters, was the assumed object of the experimenters. In view of this fact these studies on waste and repair of the body were characterised by crude and uncertain methods of control. To have met the scientific requirements of modern pharmacology the analytical methods should have taken accurate account of both intake and outgo, and where the two failed to balance the difference must have been found with demonstrable precision. My notes, embracing many pages based upon Report 88, show numerous and flagrant disregard of accurate determinations of either the intake or the outgo. Even in the instance of nitrogen, in which the effort was made to seem accurate, the variableness of the balance of nitrogen and the failure to account for such balances when demonstrated, together with the tendency in other instances to brush them aside as of no importance, show the defectiveness of the methods employed. This method of dealing with what is really the crux of the whole question is shown by the fact that when one of the subjects, a physician, insisted during the experiment that the benzoate was injuring him, he was assured by the particular referee who had charge of him and who was paid to protect the health of the people, that he—the subject, physician that he was, didn't know anything about his own symptoms. In another instance, calculated to throw light not only on method but motive underlying these forced interpretations, a subject who complained of "disturbed digestion, malaise and inaptitude for work," was waived aside with the assurance that the investigators "were unable to satisfy themselves that these symptoms were dependent on the benzoate ingested, but believed them to be due to other causes," which causes the investigators failed to indicate by so much as a hint. The statement is made that "there is no evidence derivable from data given in this table that there was any disturbance of nitrogen metabolism," while the record of one subject, at least, showed the remarkable variation of from 2.14 negative while the benzoate was being taken to 1.42 positive after its cessation. As a matter of fact, the whole report indicates a phenomenal disturbance of nitrogen metabolism for healthy people—a disturbance that unequivocally demonstrates the harmful effects of the benzoate.

### SOME RELUCTANT ADMISSIONS.

But there were certain changes in the urine indicative of modified tissue changes induced by the benzoate in the system, that were so flagrant that they could not be ignored. Thus it was shown that the benzoate taken in came out as hippuric acid—a long established fact— that the hippuric acid thrown off was relative to the benzoate taken in—a fact long accepted; that the difference between intake and output represented the residual benzoate held in the system—an obviously logical proposition; and finally, the persistence of the hippuric acid in the urine above the normal for days if not for weeks after the benzoate had been discontinued proved that a part of it remained in and consequently, under continuous usage, tended to accumulate in the system even when given under cover of a medicated diet—all of which are fundamental facts upon which the government based its original position against benzoate in food. The deleterious effect of such a persistent burden and of such an active irritant upon the kidneys is today recognised by every competent medical man in the country. Yet in spite of this fact and in spite of all the additional facts that this indubitable testimony is embodied in the report of every one of the referees, their united finding is that benzoate of soda "has not been found to exert any deleterious effect on the general health, nor to act as a poison in the general acceptation of the term." Then, as if this particular evidence might better not be ignored, but that it might be well to minimise its significance, there is a naive admission that "in some directions there were slight modifications in certain physiological processes, the exact significance of which modification is not known."

A STUDY IN EXCUSES.

That there was a constant purpose to exculpate benzoate as the cause of all functional disturbances in the subjects is apparent on almost every page. Thus, for example, the temperature of the subjects in the New Haven squad was reduced, but the fact was ignored by the referee in his summary of results. The same is true of the pulse. A persistent effort is made to emphasise the fact that the subjects increased in weight, inferentially as the result of the benzoate, while the fat producing results of a stuffed diet are as studiously withheld from mention. The board, too, has shut its eyes to the fact that the maximum of weight was realised either before the larger dosage was begun or long enough after it was discontinued to permit recovery from the effects. As a matter of fact more than 50 per cent. of the subjects used by the referees suffered more or less serious disturbances of health. More than 140 variations from the normal, obviously due to the benzoate, if interpreted by any method of fair and scientific pharmacologic determination, are studiously attributed to some other cause.

Let me give you a few examples: In the Chicago squad one subject was depressed for eight days—attributed to "cleaning the morgue." Another had depression for seven days—attributed to doing "janitor work." A lot of them developed diarrhea—attributed to "cause unknown—unless it be the cold weather," but all subjects in spite of abdominal pains were recorded as being in "excellent health." Some of them vomited, several times with blood in the vomited matter, but "no cause could be discovered."

The New Haven squad developed two cases of gastro-intestinal trouble—due to the "hot weather of a New England summer" (which seems to have the same effect in Connecticut that the cold of winter has in Illinois). One man lost his appetite—due to "hard physical work," which in Cincinnati is generally supposed to be appetising. Two had casts in the urine—due in one case to a "student rush," in the other to "a wrestling match." Numerous other instances, equally absurd, could be given from the reports under review.

### SOME THINGS ADMITTED.

Each referee seems, however, to be impressed with the importance, obviously for the sake of appearance of fairness, of admitting some offense chargeable to the benzoate. One admits that it lessens the acidity of normal urine—an acknowledged menace to health—and that it acts as a diuretic—certainly a bad thing in a regular article of diet to be consumed year after year. Another insists that normal gas formation in the digestive canal is restricted and even totally suppressed by benzoate—a serious disturbance of physiologic equilibrium. The development of certain abnormal metabolic products is frankly admitted—another evidence of profound systemic disturbance. A distinct irritant effect of benzoate on the stomach and intestines is conceded—a potent cause of dyspepsia, diarrhea and general ill health.

### MILK ADULTERATION AUTHORISED.

We shudder when we consider that, under the permission of this board's findings, the milk intended for defenseless infants may now be medicated without limit by this drug, so far as our national law is concerned. Then, when we consider the increased activity of the benzoate in acid media, we wonder why, instead of unqualified license, at least one word of warning was not uttered against its use in cider, and especially in fruit syrups now extensively consumed at soda fountains by the general public and that are being increasingly used in the sick room by convalescents struggling for health.

### SOME SECONDARY EFFECTS OF BENZOATE.

Now it would seem that if this board had been made up of pharmacologists it doubtless would have interpreted these conceded primary effects, at least somewhat in the light of their necessary after-effects. This interpretation would, of course, have been based upon the assumption that the active cause, the benzoate, was indefinitely and indiscriminately continued under the present license of the government promulgated on the advice of these referees. Recognition would have been given, as it was not given, to the fact that any diuretic that persistently lowers the acidity of the urine below normal must sooner or later induce organic diseases of the kidneys, as the precipitation of solids and the consequent formation of stones in the bladder and kidneys. It would have been pointed out, as it was not pointed out, that any antiseptic that persistently suppresses normal gas formation suppresses normal fermentation and tends to lay the foundation for permanent loss of function. It would have been mentioned, as it was not mentioned, that any irritant swallowed three times a day with persistent regularity for a long time must inevitably induce permanent organic change in the stomach and bowels. It would have been emphasised, as it was not even mentioned, that the development of abnormal metabolic products in the excretions means abnormal tissue waste and that abnormal tissue waste means inevitable impairment of strength and efficiency.

### THE SELF-CONDEMNATION OF THE REPORT.

If now we even ignore the many things that this report would conceal and confine ourselves only to the things it has been forced to reveal we can not but be astonished that the referees who framed it so far ignored the force of their own evidence as to recommend an acquittal of benzoate of soda as an offender under the law. The entire weight of their report is to the contrary effect and forces me to reply to my initial interrogatories as follows:

First—The questions submitted by the government to the referees covered the whole ground.

Second—The experiments, so far as indicated by the report, were not made by the members of the Referee Board of Scientific Experts, nor even under their constant personal supervision.

Third—The experiments themselves (a) were not conducted with sufficient care to give them scientific precision as a whole; (b) they were not sufficiently comprehensive to comprise an answer to the questions submitted by the government; (c) they were not recorded with sufficient freedom from bias to entitle them to public confidence.

Fourth—The recorded facts, taken at their face value, do not justify the conclusions embodied in the report and consequently do not justify the recommendations that the government naturally accepted in its faith in these referees to grant a license for the unlimited use of benzoate of soda in the food of the American people.

